# Nanomaterials for gas sensing and delivery

**DOI:** 10.1039/d4na90050b

**Published:** 2024-05-17

**Authors:** Run Zhang, Songjun Zeng, Rona Chandrawati

**Affiliations:** a Australian Institute for Bioengineering and Nanotechnology, The University of Queensland St. Lucia Queensland 4072 Australia r.zhang@uq.edu.au; b School of Physics and Electronics, Key Laboratory of Low-dimensional Quantum Structures and Quantum Control of the Ministry of Education, Hunan Normal University Changsha Hunan 410081 China songjunz@hunnu.edu.cn; c School of Chemical Engineering, Australian Centre for Nanomedicine (ACN), The University of New South Wales Sydney NSW 2052 Australia rona.chandrawati@unsw.edu.au

## Abstract

Run Zhang, Songjun Zeng, and Rona Chandrawati introduce the *Nanoscale Advances* themed issue ‘Nanomaterials for gas sensing and delivery’.
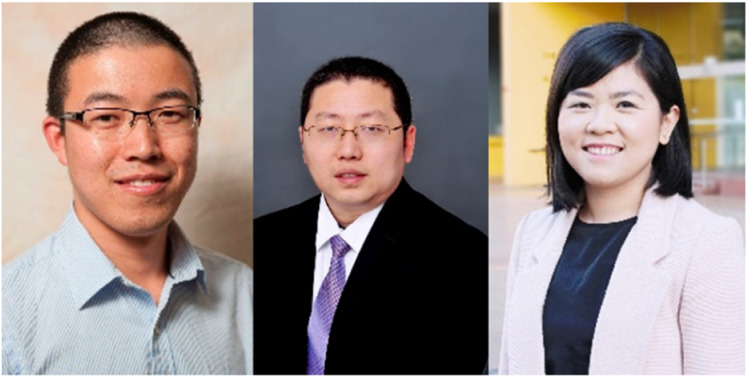

Gas molecules play an essential role in various environmental and health applications, spanning from atmospheric composition and climate regulation to biomedical diagnosis and therapies, as well as industrial processes. For instance, oxygen (O_2_), nitrogen (N_2_), carbon dioxide (CO_2_), methane (CH_4_), and ozone (O_3_) are key atmospheric components, while elevated levels of CO_2_, CH_4_, O_3_ and other greenhouse gases, such as nitrous oxide (N_2_O) resulting from increasing human activities, pose significant environmental and human health risks. Other gases, such as nitric oxide (NO), hydrogen sulfide (H_2_S), carbon monoxide (CO), and sulfur dioxide (SO_2_) are known for their high toxicity. However, recent studies have established their role as gasotransmitters in the body with important physiological functions. Therefore, effective management and responsible use of gases is essential for sustainable development and safeguarding human health and the environment.

Gas sensors are instrumental in enhancing gas management practices by providing reliable, real-time data for decision-making, risk assessment, and implementing proactive measures to protect human health and the environment. Specifically for biomedical studies, several gas molecules have been key biomarkers for various diseases, such as cardiovascular disease, cancers, and neurodegenerative disorders (*e.g.*, Alzheimer's and Parkinson's disease). Detection of these gaseous biomarkers using sensors tailored for biomedical investigations is contributing significantly to early diagnosis of these diseases and monitoring the treatment efficiency. Moreover, considering the important roles of gaseous biomolecules in disease development, recent research involves the delivery of exogenously therapeutic gases (*e.g.*, gasotransmitters, hydrogen) to the targeted diseased tissues for therapy.

Despite notable progress in gas sensing and delivery technologies, several challenges persist in their development and implementation. Selectivity, sensitivity, response time and stability are common considerations in developing advanced sensors for gas detection. In gas therapies, precise delivery of the gas to the targeted diseased tissue and the subsequent controlled release by external and internal stimulations are crucial to achieve high treatment outcomes with minimal side effects.

The last few decades have witnessed rapid progress in the development of advanced nanotechnology for various applications in medicine, environmental and life sciences, energy, and catalysis. In particular, to address the challenges in the management of gases (including noxious gas and therapeutic gas), a series of nanoscale materials with fascinating structural, physical, and chemical characteristics have been developed for gas sensing and delivery in recent years. Specifically, nanosensors with a reliable, fast, sensitive, and specific response to target gaseous molecules or vapours, are proved to be one of the most direct and effective tools for gas detection and identification. In biomedical and medicinal investigations, theranostic nanoparticles have shown great potential in shuttling drug-like gases (*e.g.* gasotransmitters, H_2_, O_2_, *etc.*) to remote diseased sites for therapeutic purposes.

*Nanoscale Advances* is an international gold open access journal, publishing high-quality papers across the breadth of nanoscience and nanotechnology. In this themed collection of *Nanoscale Advances*, we aim to provide a forum for recent trends in the rapidly evolving field of nanomaterials for gas sensing and delivery. The selection includes both review and original research articles, covering the preparation and characterization of nanomaterials (*e.g.*, graphene, quantum dots, monocrystalline, metal–organic framework, and two-dimensional nanomaterials, such as Mxene), their applications in the management of gases (*e.g.*, nitrogen dioxide), and the development of nanomaterials-based devices for gas sensing.

As Guest Editors of this themed issue, we would like to express our sincere gratitude to the authors for their high quality contributions, all anonymous reviewers who dedicated their valuable time to evaluate the submissions, and members of the *Nanoscale Advances* editorial team (especially Dr Hannah Kerr) for their guidance and support throughout the preparation of this issue.

## Supplementary Material

